# A Preclinical Systematic Review of Curcumin for Protecting the Kidney with Ischemia Reperfusion Injury

**DOI:** 10.1155/2020/4546851

**Published:** 2020-11-12

**Authors:** Zi-Hao Wang, Li-Hui Deng, Chang-Wei Chi, Hong Wang, Yue-Yue Huang, Qun Zheng

**Affiliations:** Department of Nephrology and Rheumatism Immunity, The Second Affiliated Hospital and Yuying Children's Hospital of Wenzhou Medical University, Wenzhou, China

## Abstract

Renal ischemia-reperfusion injury (RIRI) refers to a phenomenon associated with dysfunction of the kidney and tissue damage. Unfortunately, no specific drugs have been found that effectively prevent and treat RIRI. Curcumin (Cur), a polyphenol extracted from turmeric, possesses a variety of biological activities involving antioxidation, inhibition of apoptosis, inhibition of inflammation, and reduction of lipid peroxidation. Eight frequently used databases were searched using prespecified search strategies. The CAMARADES 10-item quality checklist was used to evaluate the risk of bias of included studies, and the RevMan 5.3 software was used to analyze the data. The risk of bias score of included studies ranged from 3 to 6 with an average score of 5.22. Compared with the control group, Cur significantly alleviated renal pathology, reduced blood urea nitrogen and serum creatinine levels, and improved inflammatory indexes, oxidant, and apoptosis in RIRI animal models. Despite the heterogeneity of the response to Cur in terms of serum creatinine, BUN, TNF-alpha, and SOD, its effectiveness for improving the injury of RIRI was remarkable. In the mouse model subgroup of serum creatinine, the effect size of the method of unilateral renal artery ligation with contralateral nephrectomy and shorter ischemic time showed a greater effect than that of the control group. No difference was seen in the methods of model establishment, mode administration, or medication times. The preclinical systematic review provided preliminary evidence that Cur partially improved RIRI in animal models, probably via anti-inflammatory, antioxidant, antiapoptosis, and antifibrosis activities and via improving microperfusion. ARRIVE guidelines are recommended; blinding and sample size calculation should be focused on in future studies. These data suggest that Cur is a potential renoprotective candidate for further clinical trials of RIRI.

## 1. Introduction

Renal ischemia-reperfusion injury (RIRI) refers to a phenomenon of aggravation of kidney dysfunction and tissue damage caused by reflow of blood to the kidneys [1, 2]. RIRI is one of the main causes of acute kidney injury (AKI) and acute renal failure (ARF) [1, 2] and is a common adverse pathophysiological change in patients with organ transplantation, shock, sepsis, burns, cardiovascular disease, and trauma [3]. Among patients with kidney transplantation, it is a major cause of delayed graft function in as many as 80% [4]. The incidence of in-hospital death is high for patients with RIRI in the intensive care unit who have a high probability of AKI. Patients who survive remain at high risk of developing chronic renal disorder that may evolve into end-stage renal disorder (ESRD), which also carries high economic, societal, and personal burdens [5]. Unfortunately, the possible mechanism of RIRI is still unclear and no specific drugs have been found to effectively prevent and treat RIRI. Therefore, it is imperative to seek a new treatment strategy to alleviate kidney damage in patients with RIRI.

**Figure 1:**
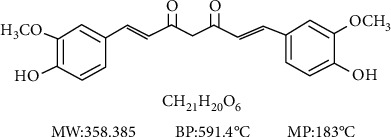
The chemical structure of Cur.

Turmeric, obtained from the rhizome of *Curcuma longa* L. (Zingiberaceae), is widely used as a spice, flavor, and colorant worldwide. Since ancient times in Asia, it has been used to prevent and treat conditions such as pain, digestive diseases, ischemic disease wounds, and gynecological problems [6]. Curcumin (Cur, C_21_H_20_O_6_, Figure 1), a polyphenol extracted from turmeric, was first isolated in 1870. Recent evidence suggests that Cur protects against ischemia injury (IR) of organs by antioxidation mechanisms, inhibiting apoptosis, inhibiting inflammatory reaction, and reducing lipid peroxidation [7]. Several studies have investigated whether supplementation with Cur improves renal pathology and renal function indexes in animal models of RIRI [4, 8, 9]. Nevertheless, scattered evidence and insufficient mechanisms have impeded the translation of laboratory results to the clinic. Systematic reviews and meta-analyses of animal studies play a pivotal role in drug development, and the clarification of physiological and pathological mechanisms could contribute to this transformation [10]. The present systematic review and meta-analysis were performed to determine the effectiveness and the mechanisms of Cur in RIRI animal models.

## 2. Materials and Methods

### 2.1. Search Strategies

Eight frequently used databases including PubMed, Cochrane Library, Embase, Wanfang database, China National Knowledge Infrastructure (CNKI), VIP database (VIP), and China Biology Medicine disc (CBM) were searched using the term “Curcumin” AND “Renal ischemia” for Cur in treatment of animal model of RIRI. The time of publication ranges from its inception to February 2020. In addition, the reference list of related studies was also searched for eligible studies.

### 2.2. Eligibility Criteria

The inclusion criteria were prespecified as follows: (1) the animal model of RIRI established by any way; (2) the treatment group accepted Cur as monotherapy at any dose and mode administration, while the control group accepted nonfunctional liquid or blank by the same dose and mode administration; (3) the primary outcome was renal pathology and/or glomerular filtration rate (GFR) and/or creatinine clearance (CCr) and/or serum creatinine (SCr) and/or blood urea nitrogen (BUN) and/or 24-hour urine protein, while the secondary outcome was the mechanisms of Cur for RIRI. The exclusion criteria were as follows: (1) not RIRI model, (2) not monotherapy, (3) no control group, and (4) duplicate publication.

### 2.3. Data Extraction

Two authors were appointed to extract the following data from included studies: (1) the surname of the first author and publication year; (2) the feature of animals including age, weight, special, male/female, and number; (3) the method of RIRI model establishment and anesthesia; (4) the dose, model administration, and duration time of the trial group and the same information of the control group; (5) the outcome index. The data of the highest dose and the result of the peak time point were extracted for analysis when multiple-dose and measurement time groups existed.

### 2.4. Quality Assessment

The CAMARADES 10-item quality checklist [2] with minor change was adopted to assess the quality of included studies. The change point is listed as follows: (F) use of anesthetic without significant intrinsic renoprotection and nephrotoxicity. Two authors independently assessed eligible studies, and the difference was settled by correspondence authors.

### 2.5. Statistical Analysis

The RevMan 5.3 software was used for statistical analysis. If meta-analysis is not applicable, the performing comparisons between groups for individual studies will be used. All data were considered as continuous data, and the combined effect size utilizes standard mean difference (SMD) or mean difference (MD) to estimate. The heterogeneity was accessed by *I*^2^ statistic. According to *I*^2^ statistic, a fixed effects model (*I*^2^ < 50%) or a random-effects model (*I*^2^ > 50%) was selected. The *P* value was considered statistically significant when the score < 0.05.

## 3. Results

### 3.1. Study Selection

**Figure 2:**
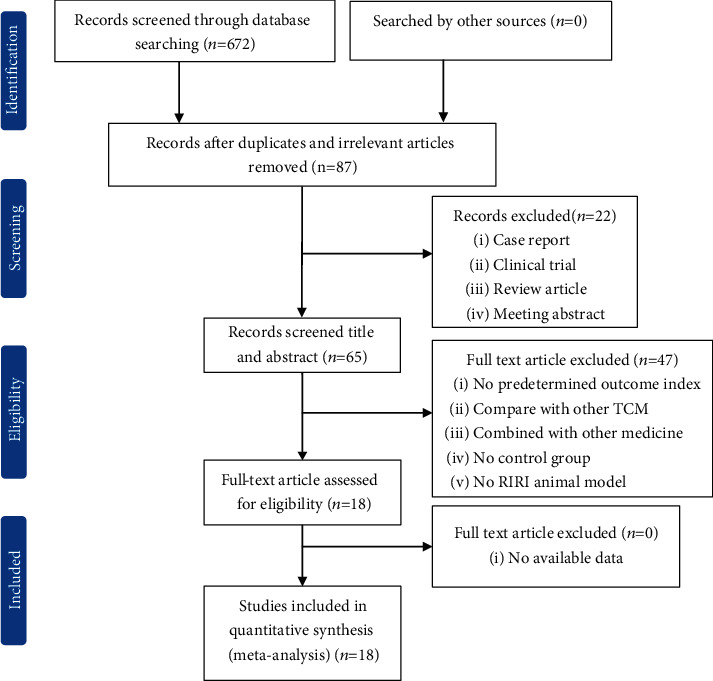
Summary of the process for identifying candidate studies.

Eighteen eligible comparison groups [4, 8, 9, 11–25] were included in the present study. The search process according to prespecified search strategies is shown in Figure 2.

### 3.2. Characteristics of Included Studies

Eleven English studies [4, 8, 9, 11–16, 24, 25] and seven Chinese studies [17–23] published from 2008 to 2019 were identified. One of the studies [22] was a non-peer-reviewed dissertation, and the remaining studies were peer-reviewed journal studies. As for animal species, SD rats were used in six studies [12, 16, 18, 20–22, 24], Wistar rats in five [4, 9, 17, 19, 23, 25], BALB/C mice in one [8], and C57/B6 mice in one [11]. Occluding renal vessel was adopted by sixteen studies [4, 8, 9, 11–19, 21, 22, 24, 25] to establish the RIRI model and sports training by two studies [20, 23] to simulate renal ischemia. Detailed information regarding the source, mode, and quality of Cur is displayed in Table 1. Five studies [4, 9, 19, 20, 23] used a dose gradient of Cur by oral administration ranging from 10 mg/kg/d to 200 mg/kg/d; seven studies [11, 12, 17, 19, 21, 24, 25] used intravenous administration ranging from 4 mg/kg/d to 100 mg/kg/d; three studies [8, 18, 22] used intraperitoneal injection ranging from 100 mg/kg/d to 200 mg/kg/d. Regarding primary outcome, renal pathology was measured in twelve studies [4, 8, 9, 11–13, 15, 18, 19, 22–25], BUN in fourteen studies [8, 9, 12, 13, 16–25], SCr in fifteen studies [4, 8, 9, 11, 16–25], and CCr in one study [13]. Mechanistic indicators and other details of the eighteen studies are summarized in Table 2.

### 3.3. Study Quality

The scores of all studies ranged from 3 to 6 with a mean score of 5.22. The methodological quality is showed in Table 3.

### 3.4. Effectiveness

#### 3.4.1. Renal Pathology

Compared with the control group, four studies showed [13, 18, 23, 24] that the Cur group had lesser degrees of macroscopic congestion, edema, and detachment of the basement membrane from glomeruli. In eleven studies [4, 8, 11–13, 15, 18, 19, 22–24], Cur mitigated turbidity and swelling and water and vacuole degeneration; the brush-like edges disappeared, and some tubular epithelial cells appeared coagulated, with necrosis of renal tubular epithelial cells. Of these, four studies [4, 8, 15, 22] utilized various renal tubular pathological scores [26, 27] to assess the renal tubular injury and found by quantification that Cur could reduce the renal tubular pathological injury.

#### 3.4.2. Renal Function Index

**Figure 3:**
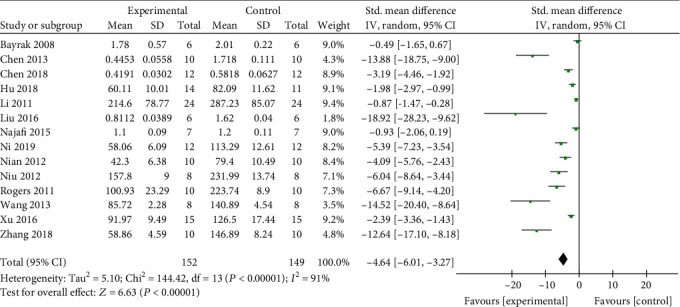
The forest plot: effects of Cur for decreasing SCr compared with the control group.

**Figure 4:**
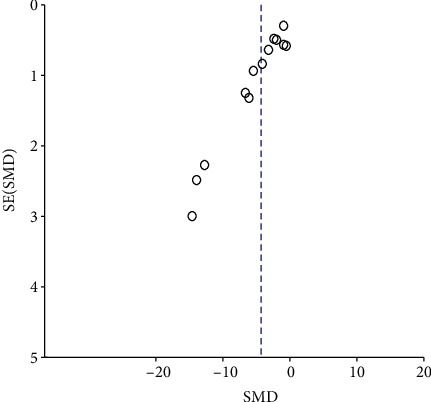
The funnel plot of SCr.

**Figure 5:**
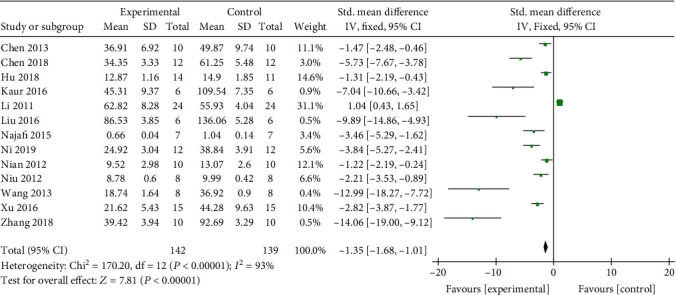
The forest plot: effects of Cur for increasing BUN compared with the control group.

Meta-analysis of 14 studies [4, 8, 9, 11, 12, 16–21, 23–25] indicated that the SCr level of the Cur groups is significantly below than that of the control groups (*n* = 301, SMD -2.08, 95% CI (-2.43-1.73), *P* < 0.00001; heterogeneity: chi^2^ = 144.42, *I*^2^ = 91%, Figure 3). This result was also showed in the dissertation [22] (*P* < 0.05). The funnel plot of the fourteen studies showed an approximately equal number of articles; however, there may have been asymmetric distribution on the central axis, indicating publication bias (Figure 4). Meta-analysis of 13 studies [8, 9, 12, 13, 16–23, 25] indicated that the serum level of BUN of the Cur groups is significantly below than that of the control groups (*n* = 281, SMD -1.35, 95% CI (-1.68, -1.01), *P* < 0.00001; heterogeneity: chi^2^ = 170.20, *I*^2^ = 93%, Figure 5). This result of BUN in the dissertation [22] showed similar conclusion (*P* < 0.05). One study [13] indicates that CCr could be increased by Cur (*P* < 0.05).

#### 3.4.3. Important Mechanism Indicator

**Figure 6:**
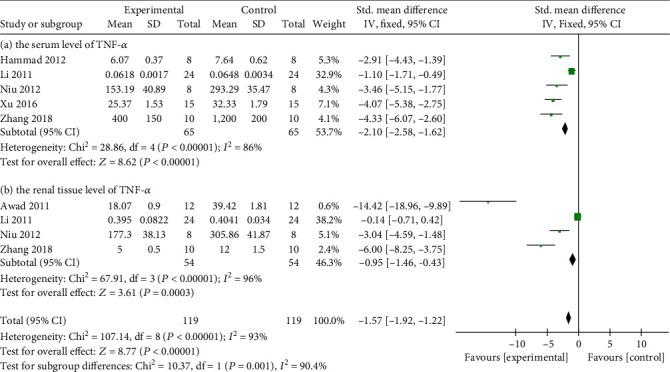
(a) The forest plot: effects of Cur for decreasing the serum level of TNF-*α* compared with the control group. (b) The forest plot: effects of Cur for decreasing the level of TNF-*α* in renal tissue compared with the control group.

**Figure 7:**
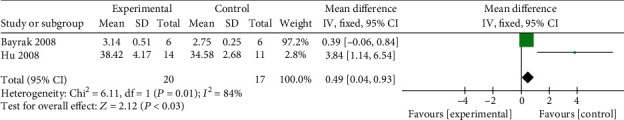
The forest plot: effects of Cur for increasing the serum level of SOD compared with the control group.

**Figure 8:**
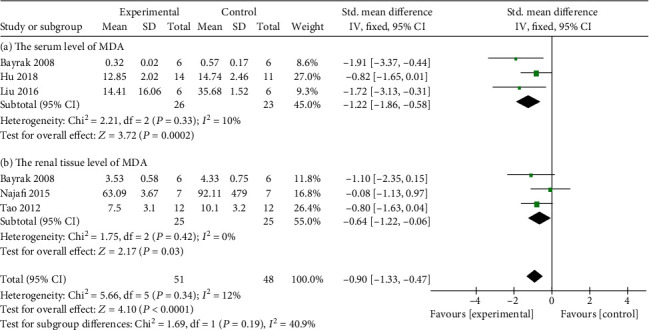
(a) The forest plot: effects of Cur for decreasing the serum level of MDA compared with the control group. (b) The forest plot: effects of Cur for decreasing the level of MDA in renal tissue compared with the control group.

In terms of anti-inflammatory mechanism, meta-analysis of five studies [12, 15, 17, 20, 21] and four studies [12, 14, 17, 20] manifested that Cur could significantly reduce the serum level of tumor necrosis factor (TNF-*α*) (*n* = 130, SMD -2.10, 95% CI (-2.58, -1.62), *P* < 0.00001; heterogeneity: chi^2^ = 28.86, *I*^2^ = 86%, Figure 6(a)) as well as the level of TNF-*α* in renal tissue (*n* = 108, SMD -0.95, 95% CI (-1.46, -0.43), *P* = 0.0003; heterogeneity: chi^2^ = 67.91, *I*^2^ = 96%, Figure 6(b)). In addition, Cur was reported to reduce the level of Interluekin-6 (IL) in renal tissue [12, 17] as well as the serum level of IL-1*β* [14, 20] and Interferon-*γ* (IFN-*γ*) [14, 20] (*P* < 0.05). In terms of antioxidant mechanism, meta-analysis of four studies showed that Cur significantly increased the serum level of superoxide dismutase (SOD) [4, 23] (*n* = 37, SMD 0.49, 95% CI (0.04, 0.93), *P* = 0.03; heterogeneity: chi^2^ = 6.11, *I*^2^ = 84%, Figure 7) as well as the level of SOD in renal tissue [4, 22] (*P* < 0.05). Serum level of Malondialdehyde (MAD) [4, 9, 23] (*n* = 49, SMD -1.22, 95% CI (-1.86, -0.58), *P* = 0.0002; heterogeneity: chi^2^ = 2.21, *I*^2^ = 10%, Figure 8(a)) was significantly reduced by Cur. Although MAD in renal tissue of dissertation [22] showed the same conclusion, it showed no difference by meta-analysis of two studies [4, 25] (*n* = 26, SMD -0.53, 95% CI (-1.52, -0.46), *P* = 0.3; heterogeneity: chi^2^ = 1.50, *I*^2^ = 33%, Figure 8(b)). In terms of antiapoptosis, two studies [22, 23] showed that Bcl-2-associated X protein/B cell lymphoma 2 (Bacl-2/Bax) was greater in the Cur group than in the control group (*P* < 0.05). Two studies [9, 14] reported that Cur could reduce the serum level of caspase-3 (*P* < 0.5).

#### 3.4.4. Subgroup Analysis

**Figure 9:**
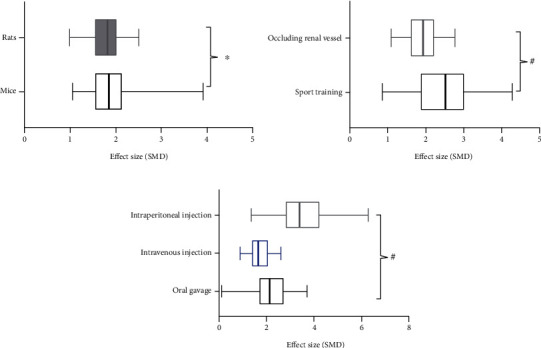
Subgroup analyses of the SCr. (a) The different effect sizes between mice and rats; (b) the different effect sizes between occluding renal vessel model group and sport training model group; (c) the different effect sizes between different mode administrations. ^∗^*P* < 0.05 between subgroups; ^#^*P* > 0.05 between subgroups.

**Figure 10:**
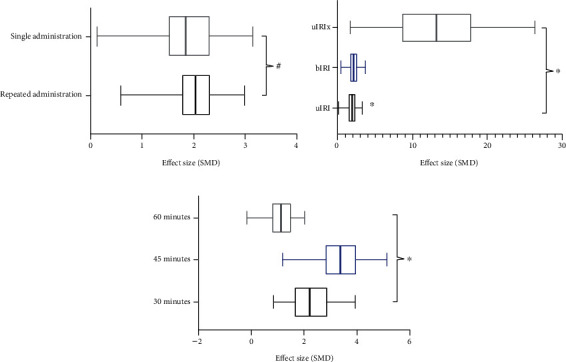
Subgroup analyses of the SCr. (a) The different effect sizes between single administration and repeated administration; (b) the different effect sizes between different occluding renal vessel model groups; (c) the different effect sizes between different ischemic times. ^∗^*P* < 0.05 between subgroups; ^#^*P* > 0.05 between subgroups.

In fourteen peer-reviewed studies, we explored potential confounding factors (including animals chosen, methods of model establishment, modes of administration, medication times, and ischemic times) that may increase the heterogeneity of outcome measures using subgroup analysis of SCr. The subgroup analysis of animal species showed that the effect size of the mouse group was better than that of rats (SMD_m_ -3.92 vs. SMD_r_ -1.89, *P* = 0.0008, Figure 9(a)) without a significant decline in heterogeneity between subgroups. No difference was seen among modeling methods (i.e., occlusion of renal vessels vs. sport training) (SMD_o_ -2.01 vs. SMD_s_ -2.49, *P* = 0.29, Figure 9(b)), diverse mode administration (including oral gavage, intravenous injection, and intraperitoneal injection) (SMD_ip_ -2.13 vs. SMD_iv_ -1.82 vs. SMD_po_ -2.07, *P* = 0.35, Figure 9(c)), and diverse medication times (including repeated administration and single administration) (SMD_r_ -2.11 vs. SMD_s_ -1.84, *P* = 0.46, Figure 10(a)). However, the effect size displayed substantial discrepancy in terms of methods of blocking blood vessels. Unilateral renal artery ligation with contralateral nephrectomy (uIRIx) with 4.7% weight showed a higher effect than did unilateral renal artery ligation (uIRI) or bilateral renal artery ligation RIRI (bIRI) (SMD_uIRIx_ -14.52 vs. SMD_bIRI_ -2.19 vs. SMD_uIRI_ -1.74, *P* < 0.00001, Figure 10(b)). Finally, we analyzed the effects of various ischemic times on the effect size of SCr and the result indicated that longer ischemia times were associated with effect size (SMD_30min_ -2.42 vs. SMD_45min_ -3.44 vs. SMD_60min_ -1.29, *P* = 0.009, Figure 10(c)).

## 4. Discussion

### 4.1. Summary of Evidence

This is the first preclinical systematic review to estimate the efficacy and possible mechanism of Cur for the RIRI animal model. The 18 moderate quality studies including 396 animals manifested that Cur alleviated renal pathological injury via multiple signaling pathways.

### 4.2. Limitations

Some limitations that may affect the accuracy of the study should be considered. First, the source of studies was only from Chinese and English databases, and this may produce selection bias. Second, the calculation of sample size and blinding outcome measurements would be pivotal for quality control of research, and this was not shown in included studies. Third, only one study [13] reported CCr, which is the most valuable clinical index for renal function. Fourth, given the fact that RIRI could not be predicted in the clinic, the preventive effect of Cur alone is insufficient. Fifth, though the sensitivity analysis and subgroup analysis were done, the high heterogeneity of curcumin for serum creatinine, BUN, TNF-alpha, and SOD cannot be ignored. Sixth, using funnel plots, there was publication bias that should be managed by expanding the sample size.

### 4.3. Implications

High-quality methodologies of studies are the cornerstones of translating animal research into clinical drug treatments for human disease [28]. Although the score (mean 5.22) by prudent assessment of included studies was better than that of most studies of traditional Chinese medicine (TCM) [29], there were limitations in terms of blinding and sample size calculation. The blinding methods in animal model establishment and outcome assessment were usually seen as technical difficulties for most studies. Group randomization after modeling, as in Guo et al. [30] and Lei et al. [31], and selecting animals randomly for outcome assessment, as in Chen et al. [8], were regarded as a good solution to overcome this problem and raise the quality of the study. A sample size calculation could avoid the waste of resources caused by oversize and the imprecision of study result by undersizing, and the specific steps could be referred to the literature [32]. In addition, the animal research reporting in vivo experiments (ARRIVE) guidelines are aimed at improving the quality of research reports by guiding complete and transparent reporting of in vivo animal research. These should be adopted in the future study management of Cur for RIRI.

Three main molding methods based on blocking blood vessels were widely utilized in the present study: uIRI in 4 studies [12, 17, 20, 22, 24], bIRI in 7 studies [4, 8, 9, 11, 16, 21, 25], and uIRIx in 2 studies [18, 19]. Subgroup analysis found that the method of uIRIx gave a higher effect size than did uIRI or bIRI (SMD_uIRIx_ -4.87 vs. SMD_bIRI_ -2.19 vs. SMD_uIRI_ -1.36, *P* < 0.00001, Figure 10(b)), suggesting that different modeling methods may be the source of high heterogeneity. Therefore, we carefully analyzed the strengths and weaknesses of these three methods and the results are summarized as follows. (1) Regarding the uIRI model, although it is easy to operate and highly repeatable, renal function as an indication of the progression of kidney injury and deterioration is difficult to estimate due to powerful compensation function of the contralateral kidney. (2) Regarding the bIRI model, it is also easy to operate and can perfectly imitate the hemodynamic changes in RIRI patients with shock, sepsis, and burns. However, the degree of renal injury is difficult to control due to the bilateral renal artery ligation. If RIRI is too severe, mice may die in the acute injury phase, and if too mild, the kidneys may fully recover and do not progress to chronic pathologies or chronic kidney disease (CKD) [33, 34]. (3) Regarding the uIRIx model, the study of Finn et al. [35] found that, if the contralateral kidney was removed prior to ischemia, the reflow of blood to the postischemic kidney would be better and conducive to recovery to preserve renal tubular structure and function. Thus, compared to the bIRI model, the uIRIx model allows for longer ischemic time to induce consistent RIRI for studying its progression to chronic pathologies with less variability and it is closer to the clinical characteristics of renal transplant patients. Compared to the uIRI model, the process of the uIRIx model is complex and changeable. The 30% death rate after 2 weeks of uIRIx by Fu et al. cannot be ignored [36]. The good news is that it allows a more accurate functional evaluation of the IRI-injured kidney at several points in time to indicate kidney injury and repair. In summary, the bIRI and uIRIx models are instrumental in monitoring renal indexes at multiple time points, but with bigger variations and significant animal loss, especially in long-term studies. The uIRI model is suitable for experiments that require a long time to observe changes of renal indexes because it can achieve the target of long-term animal survival [37]. Reviewing the included studies according to this theory, we found that the one study [18] which used the uIRI model to assess the effect of Cur for RIRI at various time points (1, 4, and 24 hours) may cause inaccurate prediction in consideration of the compensation by the contralateral kidney. We suggest that future studies need to choose the modeling method according to the specific purpose of the experimental design.

The subgroup analysis of animal species indicated that the effect size in the mouse group was better than that in the rat group (SMD_m_ -2.03 vs. SMD_r_ -1.85, *P* = 0.0006, Figure 9(a)), suggesting that diverse animals may be one of the sources of high heterogeneity. Considering high cost and low efficiency of large size for experimenters to test the initial efficacy and mechanism of the drug, rodents have become the mainstream experimental animals since the 1960s [38]. Because of the availability of transgenic models and reduced drug consumption for experimental testing, there have been more studies using mice to establish the RIRI model in the past decade [34]. Despite the advantages it possesses, it cannot be ignored that the mouse model entails greater variations, causing inconsistency in results. Thus, higher technical requirements are necessary if mice are selected as experimental animals, and the experience of mouse modeling summarized by Wei et al. [34] could be referred to in future experiments of RIRI. In addition, RIRI is a common complication for patients with infection, shock, postoperative hypoperfusion, bleeding, and dehydration; these are difficult to predict and prevent in clinical practice. However, all included studies were designed to determine whether the animals pretreated with Cur could have reduced damage of RIRI. Although the outcome was positive, it remains unknown if the effect of Cur on animals post-RIRI is similar to clinical cases of RIRI, and this may limit its clinical application. Therefore, further research designed to assess the effect of Cur for animals with post-RIRI and comparisons of the differences between pretreatment and posttreatment of Cur for RIRI are to be encouraged.

**Figure 11:**
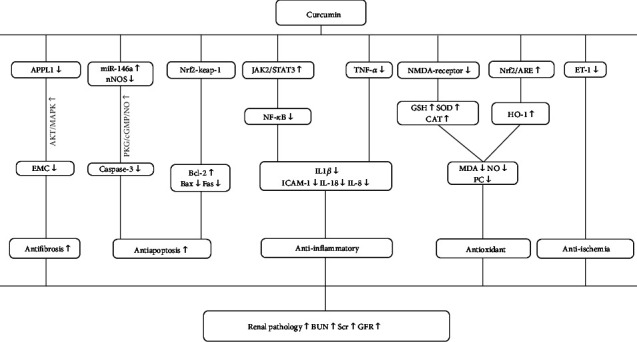
A schematic representation of osteoprotective mechanisms of Cur for RIRI.

RIRI involves several mechanisms, including mitochondrial damage, oxidative stress, calcium overload, and tissue inflammation responses [39]. (1) In the early stage of renal ischemia, neutrophils and monocytes in circulating blood are recruited by various cytokines, initiating the host's defenses. This process activates the nuclear factor kappa-B (NF-*κ*B) and further increases the release of inflammatory factors to break the proinflammatory/anti-inflammatory balance [40]. Cur was reported to alleviate renal inflammation caused by RIRI by activating the JAK2/STAT3 signaling pathway to reduce the expression of NF-*κ*B [12] by directly reducing the crucial inflammation factor TNF-*α* [9, 17, 20, 21]; it then reduces inflammatory factors including IL-1*β*, IL-8, IL-18, and intercellular cell adhesion molecule-1. (2) Oxidative stress damage is the main cause of RIRI. After vascular recanalization, vascular endothelial cells activated by reperfusion trigger the production of reactive oxygen species (ROS) and oxygen free radicals, causing oxidative stress. These processes downregulate the antioxidant enzyme system including catalase (CAT), SOD, and glutathione peroxidase (GSH-Px) [40, 41]. Cur was reported to reduce renal oxidative damage via reducing expression of N-methyl-D-aspartic acid (NMDA) receptor and increasing the expression of nuclear factor erythroid 2-related factor/heme oxygenase-1 (Nrf2/HO-1) to increase antioxidants including glutathione (GSH), SOD, and CAT and then by decreasing activity of oxidases such as MDA, nitric oxide (NO), and protein carbonyl (PC) [4, 9, 21–23]. (3) Apoptosis is a mechanism of tubular cell death in RIRI [40]. The upregulation of proapoptotic protein Bax and the downregulation of antiapoptotic protein Bcl-2 apoptosis are important processes during apoptosis when encountering ischemia [42]. Under the influence of oxidation factors, glycogen synthase kinase 3-*β* (GSK3*β*) is activated to mediate apoptosis [43]. Cur was reported to alleviate renal cell apoptosis by inhibiting activation of the PKG/cGMP/NO signaling pathway [9, 14, 22, 24] to enhance the expression of miR-146a, thereby attenuating the expression of caspase-3. It can also upregulate Bax and downregulate Bcl-2 by increasing the expression Nrf-2 [23]. (4) There were antifibrotic effects mediated by increasing the expression of adaptor protein phosphotyrosine interacting with PH domain and leucine zipper 1 (APPL1) to inhibit the AKT/MAPK signaling pathway [16] as well as a vasodilative effect by decreasing the expression of endothelin-1 (ET-1) [9]. The mechanism is summarized in Figure 11.

### 4.4. Conclusion

This preclinical systematic review provided preliminary evidence that Cur partially improves RIRI in animal models probably via anti-inflammatory, antioxidant, antiapoptosis, and antifibrosis mechanisms, as well as by improving microperfusion. The findings suggest the possibility of developing Cur as a drug for the clinical treatment of RIRI.

## Figures and Tables

**Table 1:** Information of curcumin of each study.

Study (year)	Specifications (purity)	Source	Quality control reported
Ni (2019) [24]	Dry powder	Shanghai Yuanye Biotechnology Co., Ltd.	Batch number: MO307RF
Chen (2018) [8]	NM	NM	NM
Zhang (2018) [12]	Dry powder	Sigma, St. Louis, MO	HPLC
Hu (2018) [23]	Dry powder (>99%)	Shaanxi Yuantai Biotechnology Company	Batch number: 17012571
Kaur (2016) [13]	Dry powder	Central Drug House Pvt. Ltd., India	NM
Liu (2016) [9]	Dry powder	Sigma, St. Louis, MO	HPLC
Xu (2016) [21]	Dry powder	Sigma, St. Louis, MO	HPLC
Najafi (2015) [25]	Dry powder	Sigma, St. Louis, MO	HPLC
Chen (2013) [16]	NM	NM	NM
Wang (2013) [19]	Dry powder (>90%)	Wuhan Zhongxi Instrument Daquan Company	HPLC
Hammad (2012) [15]	Dry powder	Sigma, St. Louis, MO	HPLC
Niu (2012) [20]	Dry powder (>95%)	Shanghai Ronghe Pharmaceutical Technology Development Co., Ltd.	Batch number: 110107
Nian (2012) [18]	Dry powder	Sigma, St. Louis, MO	HPLC
Tao (2012) [22]	Dry powder	Biobasic Canada Inc.	HPLC
Awad (2011) [14]	Dry powder	Sigma, St. Louis, MO	HPLC
Li (2011) [17]	Dry powder (≥90%)	Wuhan Zhongxi Instrument Daquan Company	Batch number: HB108YHSY
Rogers (2011) [11]	Dry powder	Sigma, St. Louis, MO	HPLC
Bayrak (2008) [4]	NM	NM	NM

HPLC: high-performance liquid chromatography; NM: not mentioned.

**Table 2:** Characteristics of the included studies.

Study (year)	Species (sex, *n* = experimental/control group)	Weight	Model (method)	Anesthetic	Treatment group (method to astragal sides)	Control group	Outcome index (time)	Intergroup differences
Ni (2019) [24]	SD rats (male, 10/10)	230-260 g	Block the right renal pedicle vessels for 45 minutes and then reflow for 24 hours	3.5% chloral hydrate (10 mL/kg)	By intravenous injection of 1 mL of 0.1% DMSO containing 100 mg/kg curcumin at 2 hours before establishing the model	By intravenous injection of 1 mL of 0.1% DMSO at 2 hours before establishing the model	(1) Renal pathology (2) The serum levels of *β*2-MG, UAER, BUN, and Cr (3) The expression and the transcriptional efficiency of miR-146a, nNOS, eNOS, and iNOS (4) The serum levels of NO and cGMP	(1) *P* < 0.05 (2) *P* < 0.05 (3) *P* < 0.05 (4) *P* < 0.05
Chen (2018) [8]	BALB/c mice (male, 12/12)	NM	Block the bilateral renal arteries for 30 minutes and then reflow for 24 hours	Ketamine (80 mg/kg) and xylazine (10 mg/kg)	By intraperitoneal injection of curcumin before establishing the model	By intraperitoneal injection of isovolumic NS before establishing the model	(1) The serum level of BUN and Cr (2) Kidney fibrosis (3) The expression of (ECM) protein expression (4) Western blot of APPL1 and phosphorylated Akt	(1) *P* < 0.05 (2) *P* < 0.05 (3) *P* < 0.05 (4) *P* < 0.05
Zhang (2018) [12]	SD rats (male, 10/10)	280–320 g	Block the left renal pedicle vessels for 45 minutes and then reflow for 24 hours	Pentobarbital sodium (50 mg/kg)	By intravenous injection of 60 mg/kg curcumin at 45 minutes before establishing the model	By intravenous injection of isovolumic NS before establishing the model	(1) Renal pathology (2) The serum levels of BUN and Cr (3) The serum level of TNF-*α*, IL-6, and IL-8 (4) The TNF-*α*, IL-6, and IL-8 content in renal tissue (5) Western blot of JAK2, p-JAK2, STAT3, p-STAT3, p65, and p-p65	(1) *P* < 0.01 (2) *P* < 0.05 (3) *P* < 0.05 (4) *P* < 0.05 (5) *P* < 0.05
Hu (2018) [23]	Wistar rats (male, 24/24)	218.4 ± 10.7g	8-week incremental load swimming training^[^1^]^	Ethyl ether	By oral gavage of 200 mg/kg/d curcumin during the swimming training	By oral gavage of isovolumic CMC-Na during the swimming training	(1) Renal pathology (2) The serum levels of BUN and Cr (3) The serum levels of testosterone and corticosterone (4) The serum levels of T-AOC, SOD, and MDA (5) Effect of apoptosis and Bcl-2/Bax expression in rat renal tissue (6) The expression of Nrf-2 and HO-1 in renal tissue	(1) *P* < 0.05 (2) *P* < 0.05 (3) *P* < 0.01 (4) *P* < 0.05 (5) *P* < 0.05 (6) *P* < 0.05
Kaur (2016) [13]	Wistar rats (female, 6/6)	175–225 g	Block the bilateral renal arteries for 40 minutes and then reflow for 24 hours	Ethyl ether	By oral gavage of 60 mg/kg curcumin at 60 minutes before establishing the model	By oral gavage of nothing at 60 minutes before establishing the model	(1) Renal pathology (2) CCL (3) The serum levels of BUN and UA (4) Estimation of sodium/potassium levels and macroproteinuria (5) The MPO, GSH TBARS, and SAG content in renal tissue	(1) *P* < 0.05 (2) *P* < 0.05 (3) *P* < 0.05 (4) *P* < 0.05 (5) *P* < 0.05
Liu (2016) [9]	Wistar rats (NM, 6/6)	150–200 g	Block the bilateral renal arteries for 45 minutes and then reflow for 6 hours	Pentobarbital sodium	By oral gavage of 10 mg/kg/d curcumin for 2 weeks before establishing the model	By oral gavage of isovolumic corn oil for 2 weeks before establishing the model	(1) The serum levels of BUN, Cr, and LDH (2) The serum level of MPO (3) The serum level of MDA and GSH (4) The caspase-3 and KIM-1 content in renal tissue (5) The serum levels of IL-10 and IFN-*γ*	(1) *P* < 0.001 (2) *P* < 0.001 (3) *P* < 0.001 (4) *P* < 0.001 (5) *P* < 0.001
Xu (2016) [21]	SD rats (male, 15/15)	200–250 g	Block the bilateral renal arteries for 60 minutes and then reflow for 24 hours	3% chloral hydrate (300 mg/kg)	By intravenous injection of 100 mg/kg curcumin for 5 days before establishing the model	By intravenous injection of isovolumic NS for 5 days before establishing the model	(1) The serum levels of BUN and Cr (2) The serum level of TNF-*α*, HO-1, and ICAM-1 (3) The content of MPO in renal tissue	(1) *P* < 0.01 (2) *P* < 0.01 (3) *P* < 0.01
Najafi (2015) [25]	Wistar rats (male, 7/7)	200–250 g	Block the bilateral renal arteries and veins for 30 minutes and then reflow for 72 hours	Ethyl ether	By intravenous injection of 20 mg/kg curcumin every 24 hours during 72 h reperfusion period	By intravenous injection of isovolumic NS every 24 hours during 72 h reperfusion period	(1) Renal pathology (2) The serum levels of BUN and Cr (3) The MDA and FRAP content in renal tissue (4) Leukocyte infiltration in renal tissue	(1) *P* < 0.05 (2) *P* > 0.05 (3) *P* < 0.05 (4) *P* < 0.01
Chen (2013) [16]	SD rats (NM, 10/10)	250-300 g	Block the bilateral renal arteries and veins for 45 minutes and then reflow for 3 hours	Pentobarbital sodium (35 mg/kg)	By oral gavage of 12.5 mg/kg/d curcumin for 2 days before establishing the model	By oral gavage of nothing for 2 days before establishing the model	(1) The serum levels of BUN and Cr (2) EF, LV, SV, SP, and ESPVR (3) The serum levels of CK-MB and cTnI (4) The content of MDA and TNF-*α* in heart tissue	(1) *P* < 0.05 (2) *P* < 0.05 (3) *P* < 0.05 (4) *P* < 0.05
Wang (2013) [19]	Wistar rats (male, 8/8)	220–280 g	Resect right kidney and block the left renal pedicle vessels for 45 minutes and then reflow for 24 hours	2% pentobarbital sodium (45 mg/kg)	By intravenous injection of 20 mg/kg curcumin at 30 minutes before establishing the model	By intravenous injection of nothing at 30 minutes before establishing the model	(1) Renal pathology (2) The serum levels of BUN and Cr (3) The NF-*κ*B content in renal tissue	(1) *P* < 0.05 (2) *P* < 0.05 (3) *P* < 0.05
Hammad (2012) [15]	Wistar rats (male, 8/8)	280-321 g	Block the left renal pedicle vessels for 45 minutes and then reflow for 24 hours	Ketamine hydrochloride (70 mg/kg) and pentobarbital sodium (20 mg/kg)	By oral gavage of 200 mg/kg/d curcumin for 5 days before establishing the model until the day after modeling	By oral gavage of isovolumic CMC-Na for 5 days before establishing the model until the day after modeling	(1) Renal pathology (2) GFR (3) RBF (4) UV (5) UNaV (6) FENa (7) The serum levels of TNF-*α*	(1) *P* < 0.05 (2) *P* < 0.0001 (3) *P* < 0.0001 (4) *P* > 0.05 (5) *P* > 0.05 (6) *P* > 0.05 (7) *P* < 0.05
Niu (2012) [20]	SD rats (male, 8/8)	200–280 g	Treadmill training 6 days and rest 1 day a week, circularly for 5 weeks	NM	By oral gavage of 200 mg/kg/d curcumin during the treadmill training	By oral gavage of isovolumic NS during the treadmill training	(1) The serum level of BUN and Cr (2) Renal coefficient (3) The serum level of TNF-*α*, IL-1*β* (IL-18) (4) The TNF-*α* content in renal tissue	(1) *P* < 0.05 (2) *P* < 0.01 (3) *P* < 0.05 (4) *P* < 0.01
Nian (2012) [18]	SD rats (female/male, 10/10)	200–250 g	Resect right kidney and block the right renal pedicle vessels for 45 minutes and then reflow for 2 hours	7% chloral hydrate	By intraperitoneal injection of 200 mg/kg/d curcumin for 3 days before establishing the model	By intraperitoneal injection of isovolumic NS for 3 days before establishing the model	(1) Renal pathology (2) The serum level of BUN and Cr (3) The HIF-1*α* content in renal tissue	(1) *P* < 0.05 (2) *P* < 0.05 (3) *P* < 0.05
Tao (2012) [22]	SD rats (male, 12/12)	220–260 g	Block the right renal pedicle vessels for 45 minutes and then reflow for 24 hours	10% chloral hydrate (350 mg/kg)	By intraperitoneal injection of 1 mL of 0.1% DMSO containing 100 mg/kg curcumin at 2 hours before establishing the model	By intraperitoneal injection of isovolumic 0.1% DMSO at 2 hours before establishing the model	(1) Renal pathology (2) The serum level of BUN and Cr (3) The level of MDA and SOD in renal tissue (4) The expression of Bax and Fas (5) Effect of apoptosis Bcl-2/Bax and Fas expression in rat renal	(1) *P* < 0.05 (2) *P* < 0.05 (3) *P* < 0.05 (4) *P* < 0.05 (5) *P* > 0.05
Awad (2011) [14]	SD rats (male, 12/12)	200–250 g	Block the bilateral renal content in renal tissue, arteries, and veins for 40 minutes and then reflow for 24 hours	Chloral hydrate (400 mg/kg)	By oral gavage of 100 mg/kg/d curcumin for 5 days before establishing the model	By oral gavage of isovolumic NS for 5 days before establishing the model	(1) The serum level of IL-1*β*, TGF-*β*, IL-18, IL-12, and IFN-*γ* (2) TNF-*α*, IL-1*β*, TGF-*β*, IL-18, IL-12, and IFN-*γ* (3) The TGF-*β* content in lung tissue (4) The caspase-3 content in lung tissue	(1) *P* < 0.05 (2) *P* < 0.05 (3) *P* > 0.05 (4) *P* < 0.05
Li (2011) [17]	Wistar rats (male, 24/24)	200–280 g	Block the right renal pedicle vessels for 60 minutes and then reflow for 24 hours	3% pentobarbital sodium (30 mg/kg)	By intravenous injection of 20 mg/kg curcumin at 30 minutes before establishing the model	By intravenous injection of isovolumic of NS at 30 min before establishing the model	(1) The serum level of BUN and Cr (2) Renal coefficient (3) The TNF-*α* and IL-6 content in renal tissue and serum	(1) *P* < 0.05 (2) *P* < 0.01 (3) *P* < 0.05
Rogers (2011) [11]	C57/B6 mice (male, 10/10)	NM	Block the bilateral renal arteries for 30 minutes and then reflow for 24 hours	Isoflurane	By intravenous injection of 4 mg/kg/d curcumin at 12 hours before establishing the model	By intravenous injection of 150 mL empty liposome at 12 hours before establishing the model	(1) Renal pathology (2) The serum level of urea and Cr (3) NF-*κ*B-p50 subunit in renal APC (4) Phosphorylated NF-*κ*B-p65 in renal TEC (5) TEC apoptosis (6) The gene expression of TLR4, HSP70, and TNF-*α* (7) The mRNA expression of CCL5, CCL2, and CXCL2 (8) Neutrophil infiltration in renal tissue (9) SOD mRNA expression (10) The protein expression of tyrosine nitration in renal tissue (11) The gene expression of iNOS (12) The expression of TXNIP	(1) *P* < 0.05 (2) *P* < 0.01 (3) *P* < 0.05 (4) *P* < 0.05 (5) *P* < 0.05 (6) *P* < 0.05 (7) *P* < 0.05 (8) *P* < 0.05 (9) *P* < 0.05 (10) *P* < 0.05 (11) *P* < 0.05 (12) *P* < 0.05
Bayrak (2008) [4]	Wistar rats (male, 6/6)	150–200 g	Block the bilateral renal arteries for 45 minutes and then reflow for 24 hours	Xylazine (10 mg kg^−1^) and ketamine (70 mg kg^−1^)	By oral gavage of 200 mg/kg/d curcumin for 7 days before establishing the model	By oral gavage of nothing for 7 days before establishing the model	(1) Renal pathology (2) The serum level of Cr (3) The serum level of urea and cystatin C (4) The serum level of SOD (5) The serum level of GSH-Px, MDA, NO, and PC (6) The CAT, SOD, GSH-Px, MDA, NO, and PC content in renal tissue (7) The serum level of TAC and TOS	(1) *P* < 0.05 (2) *P* < 0.05 (3) *P* > 0.05 (4) *P* > 0.05 (5) *P* < 0.01 (6) *P* < 0.05 (7) *P* < 0.001

**Table 3:** Risk of bias of the included studies.

Study	A	B	C	D	E	F	G	H	I	J	Total
Ni (2019) [24]	√	√	√			√				√	**5**
Chen (2018) [8]	√	√	√		√	√				√	**6**
Zhang (2018) [12]	√	√	√			√			√	√	**6**
Hu (2018) [23]	√	√	√			√			√	√	**6**
Kaur (2016) [13]	√	√	√			√			√	√	**6**
Liu (2016) [9]	√	√	√			√			√	√	**6**
Xu (2016) [21]	√	√	√			√				√	**5**
Najafi (2015) [25]	√	√	√			√			√	√	**6**
Chen (2013) [16]	√	√				√			√	√	**5**
Wang (2013) [19]	√	√	√			√				√	**5**
Hammad (2012) [15]	√	√	√			√			√	√	**6**
Niu (2012) [20]	√	√							√		**3**
Nian (2012) [18]	√	√	√			√			√		**5**
Tao (2012) [22]		√	√			√					**3**
Awad (2011) [14]	√	√	√			√			√	√	**6**
Li (2011) [17]	√	√	√			√					**4**
Rogers (2011) [11]	√	√	√			√				√	**5**
Bayrak (2008) [4]	√	√	√			√			√	√	**6**

Note: studies fulfilling the criteria of the following: A: peer-reviewed publication; B: control of temperature; C: random allocation to treatment or control; D: blinded induction of model (group randomly after modeling); E: blinded assessment of outcome; F: use of anesthetic without significant renoprotective activity or nephrotoxicity; G: appropriate animal model (aged, hyperlipemia or hypertensive); H: sample size calculation; I: compliance with animal welfare regulations (including three or more of the following points: preoperative anesthesia, postoperative analgesia, nutrition, disinfection, environment temperature, environment humidity, circadian rhythm, and euthanasia); J: statement of potential conflict of interest.

## Data Availability

Previously reported data were used to support this study. These prior studies and datasets are cited at relevant places within the text as references [8–10, 12–26].
